# Sex Disparities in the Processes Underlying Aging: Mitochondrial DNA Copy Number Associations with Dynapenia, 25-Hydroxyvitamin D_3_ Levels and Quality of Life in Older Adults

**DOI:** 10.3390/nu18030526

**Published:** 2026-02-04

**Authors:** Zoraida Verde, Sara Martins, Isabel Erenas-Ondategui, Maria João Santos, Celia Chicharro Miguel, Sandra Estepa Hernández, Claudia Ollauri-Ibáñez, Bárbara Oliveiros, Ana Fernández-Araque, Manuela Grazina

**Affiliations:** 1 Department of Biochemistry, Molecular Biology and Physiology, Universidad de Valladolid, Campus Duques de Soria, 42004 Soria, Spain; isabel.erenas@uva.es (I.E.-O.); celia.chicharro@uva.es (C.C.M.); sandra.estepa@uva.es (S.E.H.); claudiacasilda.ollauri@uva.es (C.O.-I.); 2 Grupo Investigación Reconocida (GIR) Farmacogenética, Genética del Cáncer, Polimorfismos Genéticos y Farmacoepidemiología, University of Valladolid, 47005 Valladolid, Spain; anamaria.fernandez@uva.es; 3 Unidad de Investigación Consolidada (UIC) de Castilla y León 387, University of Valladolid, 42004 Soria, Spain; 4 Centro de Estudios Gregorio Marañón, Fundación Ortega-Marañón, 28010 Madrid, Spain; 5 Laboratory of Mitochondrial Biomedicine and Theranostics (LBioMiT), CNC—UC-Center for Neurosciences and Cell Biology, University of Coimbra, 3000-548 Coimbra, Portugal; sara.martins@cnc.uc.pt (S.M.); mmgrazina.fmuc@gmail.com (M.G.); 6 CIBB—Center for Innovative Biomedicine and Biotechnology, University of Coimbra, 3000-548 Coimbra, Portugal; 7 Institute of Interdisciplinary Research, Doctoral Programme in Experimental Biology and Biomedicine (PDBEB), University of Coimbra, 3000-548 Coimbra, Portugal; 8 FMUC—Faculty of Medicine, University of Coimbra, 3000-548 Coimbra, Portugal; boliveiros@fmed.uc.pt; 9 Group of Environmental Genetics of Oncobiology (CIMAGO), University of Coimbra, 3000-548 Coimbra, Portugal; 10 Coimbra Institute for Clinical and Biomedical Research (iCBR), University of Coimbra, 3000-548 Coimbra, Portugal; 11 Department of Nursery, Universidad de Valladolid, Campus Duques de Soria, 42004 Soria, Spain

**Keywords:** mtDNA-CN, older adults, mineral metabolism, sex disparities, quality of life, mobility, self-care, dynapenia, hand grip strength, multimorbidity, 25(OH)D_3_

## Abstract

**Background/Objectives**: Mitochondrial dysfunction, often reflected by a decline in mitochondrial DNA copy number (mtDNA-CN) in peripheral blood cells (PMBCs), is a key hallmark of biological aging and is linked to numerous adverse health outcomes, including frailty and cardiovascular disease. Furthermore, emerging evidence suggests that vitamin D may influence mitochondrial dysfunction. This cross-sectional study aims to investigate the associations of mtDNA-CN with muscular strength, self-rated health, and serum 25-hydroxyvitamin D_3_ (25(OH)D_3_) levels in a community-dwelling elderly population. **Methods**: A total of 149 elderly outpatients (≥65 years) from Soria, Spain, were included in this cross-sectional study. Muscular strength was assessed using the hand grip strength (HGS) test, and self-rated health-related quality of life (QoL) was measured using the EuroQoL five-dimension questionnaire (EQ-5D). Genomic DNA was extracted from peripheral blood, and mtDNA-CN was quantified using quantitative real-time PCR (qPCR). Serum 25(OH)D_3_, intact parathyroid hormone (iPTH), phosphorus, calcium, albumin and other mineral metabolism markers were measured. Statistical analyses, including Spearman correlations and multivariate logistic regression, were performed to assess associations, with stratification by sex. **Results**: In the total population, a marginally significant positive correlation was observed between mtDNA copy number (mtDNA-CN) and serum 25(OH)D_3_ levels (r = 0.210; *p* = 0.010), which did not remain significant after Bonferroni correction. Among women, lower mtDNA-CN was significantly linked to muscle weakness (*p* = 0.005), mobility problems (*p* = 0.009), and a trend toward self-care difficulties (*p* = 0.016). Multivariate analysis confirmed an independent association with increased mobility impairment risk (adjusted OR = 0.983; 95% CI: 0.97–1.00; *p* = 0.009). No significant associations were observed between mtDNA-CN and dynapenia or QoL components in the male group. **Conclusions**: This study identified a marginally significant positive correlation between serum 25(OH)D_3_ levels and mtDNA-CN in the total population (r = 0.210; *p* = 0.010), which did not persist after Bonferroni correction, suggesting an exploratory link between vitamin D status and mitochondrial homeostasis in older adults. In addition, these results highlight sex-specific differences in mtDNA-CN as a potential biomarker of functional decline, particularly of mobility, in women. These findings support the idea that mtDNA-CN could serve as an integrated biomarker and that sex-specific nutrition could be used to promote healthy aging.

## 1. Introduction

Mitochondria are cytoplasmic organelles that convert dietary calories into molecular energy through a process called oxidative phosphorylation (OXPHOS) and play multiple roles in many cellular processes, including differentiation, proliferation, reprogramming homeostasis and aging [1,2,3]. Mitochondrial dysfunction has been associated with a complex clinical status, including disease susceptibility and severity [2]. Mitochondria contain their own genome, known as mitochondrial DNA (mtDNA), which encodes 37 genes [1]. Multiple copies of mtDNA are present in each mitochondrion and cells can contain up to 7000 mitochondria per cell [4].

Mitochondrial DNA copy number (mtDNA-CN) is a measure of the concentration of mtDNA load per cell. Although it is not a direct indicator of mitochondrial function, it is related to mitochondrial enzyme activity and production of adenosine triphosphate and metabolic status. mtDNA-CN is regulated in a tissue-specific manner, and unlike the nuclear genome, mtDNA is present in multiple copies per cell, the number of which is highly cell-type dependent [4,5,6].

The mtDNA-CN can be analyzed from total DNA isolated from blood and it is therefore a relatively easily attainable biomarker of mitochondrial biogenesis and function. Cells with reduced mtDNA-CN show reduced expression of fundamental complex proteins, altered cellular morphology, and lower OXPHOS [7].

It is widely accepted that mitochondria play a central role in biological aging [8]. These organelles have the capacity for self-replication and self-renewal, but they are vulnerable to structural damage and dysfunction with age through a variety of mechanisms. Peripheral blood mononuclear cells (PBMCs) are rich in mitochondria. Recently, tests of mitochondrial function in PBMCs have been proposed as a means of obtaining valid information about “general” mitochondrial health [9,10,11].

Previous research has demonstrated a correlation between lifestyle and behavioral factors and general health status and indicators in older adults [1,2]. Emerging evidence indicates a correlation between mitochondrial dysfunction and certain age-related diseases [5]. A decline in mtDNA-CN in PBMCs has been linked to several adverse health outcomes in older adults, including cardiovascular disease (CVD) [4], neurocognitive impairment [12] and chronic kidney disease [13]. Additionally, it has been suggested that the link between inflammation and health conditions may be modulated by mitochondrial dysfunction [14]. Lower mtDNA-CN has also been found to be associated with frailty, sarcopenia and all-cause mortality [15].

A small number of studies have examined the correlation between mtDNA content and quality of life (e.g., self-rated overall health) [16,17]. In addition, some disagreement appears to exist regarding the relationship between mtDNA-CN and basic demographics (sex or age) and between mtDNA-CN and self-rated health [16,17].

Emerging evidence underscores the pivotal role of vitamin D beyond its classical function in calcium homeostasis, revealing its profound influence on cellular bioenergetics and a wide number of physiological and pathological processes [18,19]. Specifically in the elderly, low levels of vitamin D have been associated with adverse skeletal effects, such as bone loss and an increased risk of fractures, with decreased skeletal muscle strength and worse quality of life (QoL) [20,21]. Another critical aspect of this expanded role of the mtDNA is the direct link between vitamin D status and mitochondrial health. Mitochondria are central to cellular function, and their integrity is largely dependent on the maintenance of mtDNA-CN. The vitamin D receptor (VDR) is now recognized as a key regulator of mitochondrial metabolism, with recent studies demonstrating its ability to translocate to mitochondria and directly interact with mtDNA [22]. This interaction is posited to regulate the transcription of mtDNA-encoded genes, thereby influencing OXPHOS and overall cellular energy production [22]. Accordingly, a compromised mtDNA-CN is a hallmark of numerous age-related and chronic diseases, including neurodegenerative disorders, cardiovascular disease, and metabolic syndrome [4,12,23]. Thus, understanding the precise mechanisms by which vitamin D modulates mitochondrial dynamics is of significant clinical and scientific importance.

The current study aimed to determine whether the mtDNA-CN is associated with dynapenia or self-rated health, as measured by a valid and well-established questionnaire (EuroQoL five-dimension questionnaire; EQ-5D), independent of basic demographic characteristics in the elderly population. As mitochondrial dysfunction has been observed in numerous chronic diseases and is associated with other non-specific symptoms which are prevalent in almost all chronic diseases, we hypothesized that mtDNA-CN, a biomarker for mitochondrial dysfunction, would be associated with health status and muscle strength. In addition, investigating the relationship between serum vitamin D levels and mtDNA-CN offers a novel pathway for understanding the systemic effects of vitamin D deficiency and provides a foundation for potential therapeutic interventions aiming to improve mitochondrial health.

## 2. Materials and Methods

### 2.1. Study Population

A cross-sectional study was conducted in this research in accordance with the Declaration of Helsinki and approved by the Health Area of Burgos and Soria Clinical Research Ethics Committee (CEIC 1446 on 28 April 2015). All subjects were randomly recruited from several primary care centers in Soria, Castilla y León National Health System (SACyL) between September 2018 and January 2019. Inclusion criteria of this trial were: (1) older individuals (age ≥65 years); (2) people who can independently detect walking speed, grip strength and muscle mass. Exclusion criteria of this trial were: (1) people who refuse to participate in the study; (2) people with impairments to complete the questionnaires; (3) malignant tumors; (4) dementia; (5) active liver disease.

For the estimating sample size, we considered a type one error (α) of 0.05 and type two error (β) of 0.20 (power = 80%), with an expected difference (d) of 5. We calculated a minimum sample size of 123 participants, based on previous data of vitamin D deficiency prevalence (plasma 25-hydroxyvitamin D3 (25(OH)D_3_) < 20 ng/mL) in older adults. A total of 158 elderly outpatients were finally enrolled in our examination program and finished a comprehensive geriatric assessment, after signing a written informed consent form.

### 2.2. Demographic and Clinical Characteristics

The sociodemographic characteristics (age, sex, institutionalization), lifestyle factors (cigarette smoking and physical activity), anthropometrics, nutrition status, medication use, falls, hospital admissions during the last year and clinical group risk (CGR) category data were collected at baseline. The CGR category is defined as a claims-based classification system for risk adjustment that assigns each individual to a single mutually exclusive risk group based on historical clinical (morbidity and chronicity) and demographic characteristics [24].

### 2.3. Questionnaires

To evaluate QoL parameters the EQ-5D questionnaire was used. This questionnaire, which has been validated in the Spanish population, is a standardized measure of health-related QoL [25]. This descriptive system evaluates the patient’s state of health in five dimensions: mobility, self-care, usual activities, pain/discomfort, and anxiety/depression. Each dimension has three levels: no problems, some problems and severe problems, where the patient has to report the most appropriate item in each dimension. The results are combined into a unique health state and then a final EQ-5D index is calculated. Moreover, the EQ-5D visual analogue scale (EQ-VAS), that can be used in a wide range of health conditions and treatments, was used as a quantitative measure of health outcome that reflected the subjects’ own judgement. It records an individual’s self-rated health on a vertical visual analogue scale, with endpoints labeled “the best health you can imagine” and “the worst health you can imagine” [26].

For the analysis of physical activity participants completed the Physical Activity Scale for the Elderly (PASE), which is a validated 12-item questionnaire that is designed to measure the level of physical activity undertaken by individuals over the age of 65. PASE assesses the types of activities typically chosen by older adults, like walking, recreational activities, exercise, housework, yard work, and caring for others [27,28].

To assess nutritional status, the Mini Nutritional Assessment (MNA) can classify older adults as well nourished, at risk for malnutrition, or malnourished. The MNA consists of 18 self-reported questions derived from the following four parameters of assessment: anthropometric assessment, general assessment, dietary assessment, and self-assessment [29,30]. We performed the full MNA for all subjects.

### 2.4. Assessment of Muscular Strength

Muscular strength was assessed using the hand grip strength (HGS) test, which was measured by a dynamometer (KYTO Digital Hand Dynamometer, Model EH101, Dongguan, China). After adjustment for hand size, three measures were performed with the dominant hand and were averaged for the analysis. Analyses of grip strength were undertaken by age and gender. The European Working Group on Sarcopenia in Older Persons defined weakness based on a grip strength less than 27 kg in men and less than 16 kg in women [31].

### 2.5. Sample Collection

Venous blood samples were drawn from each patient after fasting for 8 h or overnight. Whole blood was collected into vacutainers and either aliquoted for storage at −20 °C or processed into separate cellular and cell-free fractions within 2 h.

### 2.6. Mineral Metabolism Marker Measurement and Glomerular Filtration Rate

Bone mineral metabolism biomarker (serum total Ca^2+^, phosphorus, intact parathyroid hormone (iPTH), albumin (Alb), creatinine, and 25-hydroxyvitamin D_3_ (25(OH)D_3_)) levels were analyzed. Serum total Ca^2+^, Alb, and creatinine levels were analyzed by molecular absorption spectrometry on a Cobas 8000 c702 analyzer by Roche (Basel, Switzerland). The iPTH, phosphorus, and 25(OH)D_3_ levels were determined using electrochemiluminescent immunoassay (ECLIA) using Cobas e411 and Cobas 8000 e602 analyzers by Roche (Basel, Switzerland), respectively.

Glomerular filtration rate (GFR) was calculated using the SEMERGEN Cantabria online calculator, according to the Chronic Kidney Disease Epidemiology Collaboration (CKD-EPI) formula, based on the patient’s serum creatinine, age, sex, and ethnic group [32]. A reduced GFR below 60 mL/min was the cutoff point used to define kidney impairment in the study population.

### 2.7. Mitochondrial DNA Copy Number Measurement

Genomic DNA was extracted from peripheral blood samples using the QIAamp DNA Blood Mini Kit (Qiagen, Germantown, MD, USA) following the manufacturer’s protocol. mtDNA-CN was quantified in all samples using quantitative real-time PCR (qPCR). The specific methodology for determining mtDNA-CN, which has demonstrated high inter-assay reliability, was previously detailed by Venegas and Halberg [33].

### 2.8. Statistical Analysis

Data are expressed as mean ± standard deviation (SD) or median (Q1, Q3) for continuous variables. The distribution of the data was tested using the Kolmogorov–Smirnov test. Normally distributed data were analyzed using an independent *t*-test. Non-normally distributed data were analyzed using the Mann–Whitney test. Spearman’s tests were applied to determine the associations between continuous variables. Categorical data between two groups were compared using the chi-square test (χ^2^ test) with Fisher’s exact test. Logistic regression (LR) was used to describe and explain the relationship between dependent binary variables and independent variables. Reference groups for odds ratio (OR) calculations were low-strength group for dynapenia, no problem group for self-reported QoL parameters, and people with decreased GFR. Values of *p* < 0.05 were considered statistically significant. In the case of LR, to account for multiple testing across the seven variables, a Bonferroni correction was applied so that *p* values < 0.007 were considered statistically significant. Analysis was performed using IBM SPSS Statistics, version 28.0.

## 3. Results

### 3.1. Participant Characteristics

From September 2018 to January 2019, a total of 149 elderly adults were included (age ≥65 years). A group of nine participants were excluded from the present study for missing data on mineral metabolism markers. Compared with the excluded individuals, participants included in the present cross-sectional study were not systematically different. Table 1 provides descriptive statistics of the baseline characteristics divided by sex. The male participants presented significantly higher levels of hand grip strength, as well as a reduced incidence of falls and difficulties in daily activities (all *p* < 0.05). They also had higher levels of creatinine and lower levels of phosphorus (all *p* < 0.001).

### 3.2. Correlations Between Mitochondrial DNA Copy Number and Serum Levels of Mineral Metabolism Markers

In order to explore the importance of mtDNA-CN in mineral metabolism, the Spearman correlations between the variables were examined (Table 2). A marginally significant positive correlation between 25(OH)D_3_ and mtDNA-CN was found in the total population and also in the group of men (r = 0.210, *p* = 0.010 and r = 0.251, *p* = 0.044, respectively). Individuals with higher mtDNA-CN had higher 25(OH)D_3_ serum levels. There may be a sex bias. Consequently, a negative correlation between iPTH and mtDNA-CN was observed in males (r = −0.258, *p* = 0.039). On the other hand, albumin levels and mtDNA-CN were correlated in females (r = −0.243, *p* = 0.026).

### 3.3. Association Between Mitochondrial DNA Copy Number and Dynapenia, Quality of Life and Glomerular Filtration

A subgroup of analyses were performed according to sex. Among the female group, lower mtDNA-CN was associated with muscle weakness (*p* = 0.005) (Table 3a). In addition, it was observed that women with any problem with mobility or self-care presented lower levels of mtDNA-CN (*p* = 0.009 and *p* = 0.016, respectively). From the univariate regression analysis mtDNA-CN was associated negatively with the risk of having any problem with mobility (OR, 0.987; 95% CI, 0.98–1.00; *p* = 0.022; Table 4). This association remained, showing a trend toward association after adjustment for potential confounders (adjusted OR, 0.983; 95% CI, 0.97–1.00; *p* = 0.009; Table 4). We also observed a tendency between self-care and mtDNA-CN (*p* = 0.068), which remained marginal after multivariate analysis (*p* = 0.067).

In males, no statistically significant association between mtDNA-CN and dynapenia or QoL was found (Table 3b and Table 4). On the other hand, there was a marginally significant association between mtDNA-CN and problems with GFR (*p* = 0.031; Table 3b).

However, in this group, we confirmed that older age was significantly associated with the incidence of dynapenia or any mobility-related problems (see Table 4).

## 4. Discussion

The aging process is characterized by a series of interrelated cellular mechanisms, notably chronic low-grade inflammation and mitochondrial dysfunction [34]. These foundational processes are hypothesized to play a significant role in the etiology and progression of age-related alterations, including sarcopenia. However, given the multifactorial complexity of this condition, the precise contribution of each underlying factor remains to be fully elucidated. The mtDNA integrity, encompassing both quality and mtDNA-CN, is directly relevant to two established hallmarks of aging: genomic instability and mitochondrial dysfunction. Specifically, mtDNA-CN may serve as a quantitative biomarker reflecting mitochondrial function and content [7]. Cellular aging is intrinsically linked to an increase in oxidative stress, a state that subjects the unprotected mitochondrial genome to substantial damage from reactive oxygen species (ROS). The chronic accumulation of this mtDNA damage, and the resultant decline in mtDNA-CN, is strongly associated with the aging process. This reduction is not only a marker of aging but may also be causally linked to the development of age-related chronic diseases and the variability in health status observed within the elderly population [16,17]. Consequently, it is plausible to hypothesize that mtDNA-CN is inversely associated with chronological age, although current literature on this direct correlation remains inconclusive.

In our cohort, we did not find any correlation between mtDNA-CN and age, probably because we only included older adults and did not include any young participants for comparison. Our cohort was composed of individuals aged 65 to 94. Additionally, most previous studies indicating an age-related decline in mtDNA were either longitudinal or analyzed different generations within the same family [35,36].

A few epidemiologic studies have examined the association between mtDNA-CN and mineral metabolism markers [23,37]. The status of vitamin D could influence the oxidative capacity of mitochondria in skeletal muscle. Vitamin D supplementation and regular exercise training may increase mitochondrial fusion and total antioxidant capacity, thereby controlling oxidative stress [12]. Our findings of a marginally significant positive correlation between 25(OH)D_3_ levels and mtDNA-CN in PBMCs and are consistent with previous studies. Both Kim and Lee (2012) and Kim et al. (2012) reported similar positive associations between serum 25(OH)D_3_ and mtDNA-CN measured in circulating cells [23,37]. In the first study, higher mtDNA-CN and vitamin D levels were associated with increased femoral neck bone mineral density (BMD), and mtDNA-CN remained independently associated with osteoporosis after adjusting for vitamin D [37]. In the second, women with metabolic syndrome had significantly lower mtDNA-CN and lower vitamin D levels, and mtDNA-CN retained an independent association with the presence of the syndrome [23]. These findings support the physiological relevance of vitamin D in regulating mitochondrial homeostasis in circulating cells. Future studies should validate this association in larger, independent cohorts with adequate power to detect modest correlations, using pre-specified subgroup analyses by sex and modern multiple-testing corrections.

In humans, inadequate or deficient vitamin D status and skeletal muscle mitochondrial dysfunction are co-associated with compromised muscle strength. Beyond its established role in calcium and phosphorus homeostasis and the maintenance of skeletal health, vitamin D is increasingly recognized for its pleiotropic effects during the aging process [38]. Specifically, the active vitamin D_3_ metabolite is essential for these classic endocrine functions. While vitamin D_3_ and its metabolites exert significant clinical effects on muscle function, the precise biochemical mechanisms underlying this relationship remain to be fully elucidated [39].

The existence of crosstalk between mineral metabolism and mitochondrial dysfunction in muscle wasting represents a novel and potentially relevant field of research. Limited published literature has investigated the direct relationship between perceived health status and mtDNA-CN. However, existing evidence suggests an association between lower mtDNA-CN and poorer health outcomes, including diminished self-rated health, reduced lean muscle mass, and compromised physical performance [16,40,41,42].

The current study supports that women with dynapenia, defined as the age-related decline in muscle strength independent of muscle mass, exhibit significantly lower mtDNA-CN in peripheral blood. This finding aligns with established literature, which has consistently linked reduced mtDNA-CN (a biomarker of mitochondrial quantity and genomic stability) with sarcopenia and decreased muscle strength [41,42]. Mitochondrial dysfunction has been proposed to play a central role in the development of dynapenia, given that mitochondria are the primary producers of ATP, which is vital for muscle contraction. Furthermore, a key finding within our female cohort was the significant association between lower mtDNA-CN and reported problems in the “mobility” and “self-care” dimensions of the EQ-5D questionnaire. The EQ-5D is a validated instrument used to measure perceived health status, with these two dimensions specifically assessing an individual’s ability to move around and perform personal hygiene and daily tasks [25]. To our knowledge, this is the first study to use a validated health-related quality of life tool, such as the EQ-5D, to quantify the relationship with mtDNA-CN. Dynapenia and reduced muscle strength significantly limit an individual’s ability to perform daily activities and compromise their autonomy and independence. Numerous studies have conclusively demonstrated that sarcopenia and muscle weakness are associated with poorer QoL, impacting key areas such as mobility, daily activities, and the perception of pain and discomfort [43,44]. Consequently, the relationship observed between lower mtDNA-CN and a worse EQ-5D score may be interpreted as the result of a mitochondrial dysfunction that precipitates a decline in muscle strength, which ultimately translates into a less favorable perceived health experience.

Alongside mitochondrial remodeling, the age-related decrease in steroid sex hormone levels in both women and men has been suggested as a key driver of sarcopenia [45,46]. Indeed, these hormones can modulate distinct signaling pathways in skeletal muscle via their receptors, but the precise outcomes and sex disparities are not fully understood, particularly with regard to skeletal muscle aging [47]. This sex specificity also appears to be present in the mitochondria, with evidence suggesting that older females have lower OXPHOS complex function than older males [48,49]. The mechanisms behind the effect of sex on the development of sarcopenia and age-related mitochondrial remodeling in skeletal muscle remain to be fully uncovered [50].

In recent years, research on the relationship between mitochondrial function and kidney disease has increased gradually. Observational studies have reported controversial results regarding the association between the mtDNA-CN and renal function [13]. We found a marginally significant association between mtDNA-CN and problems in GF in males.

The exact pathophysiological mechanisms linking mitochondrial dysfunction to renal impairment remain unclear. However, the kidney’s prodigious metabolic demand strongly implicates mitochondria, as it ranks second only to the heart in mitochondrial density and oxygen consumption [51], consuming approximately 7% of the body’s daily ATP expenditure [52]. Mitochondria are fundamental for sustaining the homeostatic functions of crucial cell types, notably renal tubular epithelial cells and podocytes. A reduction in mtDNA-CN, which serves as a metric of mitochondrial integrity, compromises the capacity for OXPHOS and subsequent ATP synthesis [53]. Furthermore, mitochondrial damage exacerbates renal pathology by fostering conditions of chronic oxidative stress, promoting inflammation, and disrupting cellular quality control mechanisms like autophagy. Therefore, in view of the association between mtDNA-CN and GFR, predicting the progression of kidney injury by detecting changes in blood mtDNA-CN could be a promising approach to assess renal function loss.

The existing discrepancies observed across studies regarding mtDNA-CN and its health correlates may be attributed to several methodological and demographic factors. These include geographic origins (ethnic features) that are correlated with specificities in mtDNA variants, limited sample sizes, variations in environmental conditions, differences in cohort stratification, and the inclusion of a narrow age range of elderly participants. Furthermore, reported associations may differ based on the biological material investigated (e.g., specific tissues or cell types) or the technical variability introduced by the methods used to determine mtDNA-CN, particularly concerning the efficiency of mtDNA extraction and recovery.

It is important to acknowledge several methodological limitations in the current study. Firstly, the cross-sectional design restricts our ability to establish causal relationships or determine the temporal sequence of the observed associations between mtDNA-CN and dynapenia. Secondly, the use of PBMCs as an alternative cellular source, rather than obtaining mtDNA-CN measurements directly from muscle tissue via biopsy, means that our measurements may serve as a proxy for functional mitochondria and may not reflect the mtDNA status in the target skeletal muscle. Thirdly, while the overall cohort (N = 149) was adequately powered to detect an effect of r = 0.25, the lack of significant findings in the sex-specific analyses may be attributed to a significant loss of statistical power.

Finally, another key limitation of our cross-sectional design is reverse causality, whereby poorer health status or dynapenia may precede and cause lower mtDNA-CN rather than mtDNA-CN driving functional decline. Existing findings suggest a potential decline in mtDNA replication with age, which may be associated with age-related physiological changes. In the evolving field of personalized medicine and precision therapy, identifying individual differences in mtDNA-CN could enable the early detection of disease and the development of personalized treatment strategies. Furthermore, investigating mtDNA-CN variation as a prospective therapeutic intervention for specific conditions is set to become an innovative future strategy. Changes in mtDNA-CN have the capacity to function as both a consequence of and a driver for disease progression, encapsulating a complex biological principle that encompasses fundamental cellular processes and a wide array of clinical ramifications [54]. Prospective evidence supports the idea that lower mtDNA-CN predicts mortality, but baseline inflammation and co-morbidities explain much of this association. This suggests that health status influences mtDNA damage as the primary pathway [55]. Nevertheless, longitudinal studies measuring mtDNA-CN trajectories are essential to establish temporality and quantify bidirectional contributions.

Despite certain limitations, the current study possesses several significant strengths that enhance the robustness and novelty of its findings. Crucially, our analysis employed stratification by sex, which allowed us to identify unique and clinically relevant associations specifically within the female cohort. Furthermore, the inclusion of a well-characterized population aged 65 to 94 years provides valuable data focused on the critical elderly demographic. A key methodological strength lies in being the first study, to our knowledge, to use a validated, standardized instrument, the EQ-5D questionnaire, to quantify the association between mtDNA-CN and self-reported health-related QoL dimensions, providing an objective measure for interpreting the functional consequences of mitochondrial integrity.

Research increasingly underscores the significant role of sex disparities in skeletal muscle aging, highlighting that mitochondrial aging is a sex-specific process possibly mediated by mitochondrial estrogen receptor signaling. This necessity has led Moreira-Pais and colleagues to emphasize the urgent need for including both sexes in aging research. Such inclusion is vital to fully understand the spectrum of age-induced alterations and to enable sex-specific diagnostic and management strategies for skeletal muscle diseases like sarcopenia [50].

## 5. Conclusions

In conclusion, the findings of this study identified a marginally statistically significant positive correlation between circulating 25(OH)D_3_ levels and mtDNA-CN in peripheral blood cells within the elderly population. This association highlights a relevant physiological link between mineral metabolism and mitochondrial homeostasis during aging. Furthermore, within the female cohort, we demonstrate a trend toward an inverse relationship between mtDNA-CN and the clinical manifestation of dynapenia, alongside self-reported problems in the mobility and self-care dimensions of the EQ-5D questionnaire. Multivariate analysis confirmed an independent association with increased mobility impairment risk. To our knowledge, this is the first study to quantitatively link mtDNA-CN with health-related QoL assessed via a validated instrument. Specifically, the sex-specific association between lower mtDNA-CN, dynapenia, and diminished QoL (mobility/self-care) in women but not men can be supported by compelling scientific literature focusing on mitochondrial sex differences and the distinct roles of hormones in muscle aging. The recognition of sex-specific players involved in age-related skeletal muscle impairment will enable the development of preventive and therapeutic interventions tailored to the sex of each patient towards improved chances of survival and QoL. These results suggest that mtDNA-CN may serve as an integrated biomarker reflecting the collective impact of compromised mineral status, muscle strength decline, and resulting functional limitation on overall QoL in older adults. Future longitudinal studies are warranted to establish the causal direction of these relationships and to explore the therapeutic potential of interventions targeting vitamin D status or mitochondrial integrity to mitigate age-related functional decline.

## Figures and Tables

**Table 1:** Basic characteristics, mineral metabolism biomarkers and mitochondrial DNA copy number, stratified by sex.

	Total (N = 149)	Females (N = 84)	Males (N = 65)	*p*
**Age (Mean ± SD)**	76.28 ± 7.09	75.68 ± 7.06	77.05 ± 7.11	0.240
**BMI (Mean ± SD)**	27.67 ± 4.09	27.62 ± 4.22	27.74 ± 3.94	0.854
**Living situation N (%)**				
Alone	46 (30.9)	24 (28.6)	22 (33.9)	0.489
Together	103 (69.1)	60 (71.4)	43 (66.1)	
**CGR N (%)**				
G0	56 (37.6)	31 (36.9)	25 (38.5)	0.307
G1	50 (33.5)	33 (39.3)	17 (26.1)	
G2	28 (18.8)	13 (15.5)	15 (23.1)	
G3	15 (10.1)	7 (8.3)	8 (12.3)	
**Falls N (%)**				
No	130 (87.3)	67 (78.8)	63 (96.9)	**0.002**
Yes	19 (12.7)	17 (21.2)	2 (3.1)	
**Emergency visits N (%)**				
No	134 (89.9)	76 (90.5)	58 (89.2)	0.802
Yes	15 (10.1)	8 (9.5)	7 (10.8)	
**Smoker N (%)**				
No	144 (96.6)	83 (98.8)	61 (93.9)	0.095
Yes	5 (3.4)	1 (1.2)	4 (6.1)	
**Diabetes mellitus N (%)**				
No	133 (89.3)	77 (91.7)	56 (86.1)	0.281
Yes	16 (10.7)	7 (8.3)	9 (13.9)	
**PASE (Mean ± SD)**	278.69 ± 169.04	284.89 ± 168.75	270.68 ± 170.39	0.544
**Grip test right (Mean ± SD)**	38.02 ± 21.03	32.05 ± 16.51	45.74 ± 23.70	**<0.001**
**Grip test left (Mean ± SD)**	35.24 ± 21.96	30.56 ± 20.65	41.29 ± 22.28	**0.002**
**Dynapenia N (%)**				
Low	31 (20.8)	15 (17.9)	16 (24.6)	0.314
Not low	118 (79.2)	69 (82.1)	49 (75.4)	
**EQ-5D (Mean ± SD)**	0.90 ± 1.08	0.96 ± 1.43	0.83 ± 0.16	0.240
**Mobility N (%)**				
No problem	112 (75.2)	62 (73.8)	50 (76.9)	0.663
Problem	37 (24.8)	22 (26.2)	15 (23.1)	
**Self-care N (%)**				
No problem	142 (95.3)	79 (94.1)	63 (96.9)	0.411
Problem	7 (4.7)	5 (5.9)	2 (3.1)	
**Daily activities N (%)**				
No problem	136 (91.3)	73 (86.9)	63 (96.9)	**0.032**
Problem	13 (8.7)	11 (13.1)	2 (3.1)	
**Pain N (%)**				
No problem	78 (52.3)	43 (51.2)	35 (53.8)	0.748
Problem	71 (47.7)	41 (48.8)	30 (46.2)	
**Anxiety/depression N (%)**				
No problem	101 (67.8)	52 (61.9)	49 (75.4)	0.081
Problem	48 (32.2)	32 (38.1)	16 (24.6)	
**EQ-VAS (1–100) (Mean ± SD)**	72.95 ± 16.44	71.73 ± 18.38	74.54 ± 13.51	0.452
**VAS (1–10) (Mean ± SD)**	2.69 ± 2.53	2.76 ± 2.51	2.59 ± 2.58	0.606
**MNA (Mean ± SD)**	13.76 ± 1.88	13.87 ± 1.85	13.61 ± 1.93	0.419
**mtDNA (Mean ± SD)**	138.82 ± 62.45	145.18 ± 65.30	130.61 ± 58.01	0.184
**Creatinine (ng/mL) (Mean ± SD)**	0.95 ± 0.26	0.84 ± 0.19	1.09 ± 0.27	**<0.001**
**Calcium (ng/mL) (Mean ± SD)**	9.46 ± 0.36	9.51 ± 0.40	9.39 ± 0.28	0.084
**Phosphorus (ng/mL) (Mean ± SD)**	3.24 ± 0.47	3.36 ± 0.44	3.09 ± 0.46	**<0.001**
**Albumin (ng/mL) (Mean ± SD)**	4.41 ± 0.25	4.38 ± 0.26	4.45 ± 0.24	0.162
**PTH (ng/mL) (Mean ± SD)**	65.98 ± 29.44	68.97 ± 32.54	62.06 ± 24.49	0.405
**25(OH)D_3_ (total) (ng/mL) (Mean ± SD)**	18.67 ± 9.29	18.47 ± 9.04	18.93 ± 9.68	0.723
**GFR (mL/min/1.73 m^2^) (Mean ± SD)**	69.55 ± 16.26	70.81 ± 17.03	67.92 ± 15.19	0.379
Decreased	41 (27.5)	22 (26.2)	19 (29.2)	0.608
Normal	108 (72.5)	62 (73.8)	46 (70.8)	

SD: Standard Deviation; BMI: body mass index; CGR: clinical group risk; PASE: Physical Activity Scale for the Elderly; EQ-5D: EuroQoL Five-Dimension Questionnaire; EQ-VAS: EQ-5D Visual Analogue Scale; VAS: Visual Analogue Scale; MNA: Mini Nutritional Assessment; mtDNA-CN: mitochondrial DNA copy number; iPTH: intact parathyroid hormone; 25(OH)D_3_: 25-hydroxyvitamin D_3_; GFR: Glomerular Filtration Rate. Dynapenia was considered low under 16 kg for women and under 27 kg for men. Statistically and marginally significant values are in bold.

**Table 2:** Spearman Rank order correlation between mitochondrial DNA Copy Number and mineral metabolism markers.

	mtDNA-CN r (*p*)		
	Total (N = 149)	Females (N = 84)	Males (N = 65)
**Age**	−0.051 (0.533)	0.009 (0.936)	−0.069 (0.583)
**Creatinine (ng/mL)**	−0.128 (0.121)	−0.045 (0.687)	−0.157 (0.213)
**Calcium (ng/mL)**	0.022 (0.788)	−0.060 (0.589)	0.072 (0.569)
**Phosphorus (ng/mL)**	−0.018 (0.829)	−0.057 (0.607)	−0.081 (0.520)
**Albumin (ng/mL)**	−0.120 (0.146)	−0.243 **(0.026)**	0.051 (0.686)
**iPTH (ng/mL)**	−0.117 (0.156)	−0.004 (0.969)	−0.258 **(0.039)**
**25(OH)D_3_ (total) (ng/mL)**	0.210 **(0.010)**	0.185 (0.092)	0.251 **(0.044)**
**GFR (mL/min/1.73 m^2^)**	0.122 (0.138)	0.060 (0.586)	0.163 (0.195)

r: Spearman coefficient: mtDNA-CN: mitochondrial DNA copy number; iPTH: intact parathyroid hormone; 25(OH)D_3_: 25-hydroxyvitamin D_3_; GFR: Glomerular Filtration Rate. Statistically and marginally significant values are in bold.

**Table 3:** (**a**) Univariate analysis of baseline characteristics in women regarding dynapenia, quality of life dimensions and renal function. (**b**) Univariate analysis of baseline characteristics in men regarding dynapenia, quality of life dimensions and renal function.

	Dynapenia			Mobility			Self-Care			Daily Activities			**Pain/Discomfort**			**Anxiety/Depression**			**GFR**		
	Low	Not Low	*p*	No Problem	Problem	*p*	No Problem	Problem	*p*	No Problem	Problem	***p***	**No Problem**	**Problem**	***p***	**No Problem**	**Problem**	***p***	**1**	**2**	***p***
	Mean ± SD	Mean ± SD		Mean ± SD	Mean ± SD		Mean ± SD	Mean ± SD		Mean ± SD	Mean ± SD		**Mean ± SD**	**Mean ± SD**		**Mean ± SD**	**Mean ± SD**		**Mean ± SD**	**Mean ± SD**	
(**a**)																					
**Age**	76.80 ± 6.39	75.43 ± 7.22	0.423	75.40 ± 6.62	76.45 ± 8.30	0.706	75.89 ± 7.16	72.40 ± 4.34	0.327	75.66 ± 6.97	75.82 ± 7.97	0.979	75.14 ± 7.21	76.24 ± 6.94	0.415	75.00 ± 7.07	76.78 ± 7.01	0.205	79.55 ± 8.15	74.31 ± 6.13	**0.004**
**BMI**	26.39 ± 4.36	27.88 ± 4.17	0.197	27.59 ± 4.09	27.70 ± 4.67	0.976	27.63 ± 3.92	27.46 ± 8.36	0.450	27.54 ± 4.00	28.13 ± 5.69	0.874	27.74 ± 4.00	27.49 ± 4.48	0.775	27.42 ± 3.88	27.94 ± 4.77	0.662	28.02 ± 4.39	27.47 ± 4.18	0.576
**PASE**	316.48 ± 161.13	278.02 ± 170.71	0.267	323.86 ± 170.59	175.06 ± 104.38	**<0.001**	289.62 ± 168.27	210.20 ± 176.93	0.185	293.58 ± 170.98	227.24 ± 147.19	0.201	333.81 ± 173.91	233.58 ± 148.52	**0.006**	330.80 ± 175.12	210.29 ± 128.48	**<0.001**	250.81 ± 159.28	296.98 ± 171.59	0.329
**Grip test right**	14.87 ± 5.19	35.78 ± 15.75	**<0.001**	32.15 ± 16.89	31.75 ± 15.78	0.927	31.53 ± 16.39	40.20 ± 18.17	0.219	32.15 ± 16.61	31.36 ± 16.63	0.974	32.65 ± 17.81	31.41 ± 15.23	0.911	31.35 ± 17.55	33.19 ± 14.87	0.321	30.36 ± 15.50	32.65 ± 16.94	0.733
**Grip test left**	23.13 ± 36.76	32.17 ± 15.08	**<0.001**	31.32 ± 22.46	28.41 ± 14.60	0.729	30.11 ± 20.77	37.60 ± 19.27	0.274	30.47 ± 21.29	31.18 ± 16.58	0.666	29.80 ± 16.82	31.35 ± 24.21	0.957	29.09 ± 16.41	32.95 ± 26.25	0.641	32.39 ± 30.59	29.91 ± 16.02	0.665
**EQ-5D**	1.66 ± 3.36	0.80 ± 0.17	0.891	1.06 ± 1.65	0.66 ± 0.13	**<0.001**	0.98 ± 1.47	0.51 ± 0.16	**<0.001**	0.83 ± 0.15	1.81 ± 3.98	**0.002**	1.22 ± 1.97	0.68 ± 0.12	**<0.001**	1.12 ± 1.80	0.69 ± 0.12	**<0.001**	1.33 ± 2.79	0.82 ± 0.17	0.146
**EQ-VAS (1–100)**	69.67 ± 20.57	72.17 ± 18.00	0.570	75.89 ± 15.54	60.00 ± 20.93	**<0.001**	73.16 ± 16.43	49.00 ± 32.48	0.075	73.77 ± 16.79	58.18 ± 23.27	**0.022**	79.88 ± 13.25	63.17 ± 19.23	**<0.001**	75.67 ± 17.12	65.31 ± 18.79	**0.007**	71.59 ± 21.68	71.77 ± 17.25	0.533
**VAS (1–10)**	3.43 ± 3.25	2.62 ± 2.32	0.478	2.40 ± 2.37	3.77 ± 2.65	**0.032**	2.77 ± 2.51	2.60 ± 2.70	0.898	2.84 ± 2.56	2.27 ± 2.20	0.550	1.08 ± 1.36	4.52 ± 2.21	**<0.001**	2.33 ± 2.50	3.47 ± 2.40	**0.021**	2.73 ± 2.69	2.77 ± 2.46	0.833
**MNA**	13.20 ± 2.08	14.01 ± 1.79	0.147	13.94 ± 1.88	13.68 ± 1.81	0.478	13.89 ± 1.84	13.60 ± 2.30	0.826	13.97 ± 1.83	13.18 ± 1.94	0.169	14.30 ± 1.47	13.41 ± 2.11	0.063	14.13 ± 1.79	13.44 ± 1.90	0.086	13.05 ± 2.21	14.16 ± 1.63	**0.036**
**mtDNA-CN**	121.75 ± 97.87	150.27 ± 55.55	**0.005**	155.01 ± 68.68	117.48 ± 45.46	**0.009**	148.31 ± 66.07	95.79 ± 11.82	0.016	148.50 ± 67.55	123.13 ± 43.96	0.147	148.86 ± 55.76	141.33 ± 74.53	0.306	145.45 ± 71.80	144.74 ± 54.20	0.733	144.70 ± 53.46	145.35 ± 69.42	0.722
**Creatinine (ng/mL)**	0.87 ± 0.21	0.83 ± 0.18	0.532	0.83 ± 0.19	0.84 ± 0.18	0.699	0.84 ± 0.19	0.85 ± 0.14	0.581	0.83 ± 0.19	0.89 ± 0.20	0.330	0.82 ± 0.19	0.86 ± 0.19	0.230	0.82 ± 0.18	0.86 ± 0.20	0.469	1.05 ± 0.20	0.76 ± 0.11	**<0.001**
**Calcium (ng/mL)**	9.55 ± 0.51	9.51 ± 0.38	0.774	9.47 ± 0.43	9.63 ± 0.32	0.057	9.50 ± 0.41	9.70 ± 0.23	0.205	9.50 ± 0.42	9.62 ± 0.24	0.236	9.47 ± 0.39	9.56 ± 0.42	0.336	9.48 ± 0.43	9.57 ± 0.36	0.235	9.51 ± 0.33	9.51 ± 0.43	0.963
**Phosphorus (ng/mL)**	3.29 ± 0.47	3.37 ± 0.44	0.643	3.30 ± 0.43	3.50 ± 0.44	0.090	3.35 ± 0.45	3.54 ± 0.11	0.227	3.33 ± 0.46	3.54 ± 0.23	0.120	3.34 ± 0.40	3.38 ± 0.48	0.666	3.33 ± 0.46	3.39 ± 0.42	0.351	3.43 ± 0.37	3.33 ± 0.46	0.621
**Albumin (ng/mL)**	4.39 ± 0.28	4.38 ± 0.25	0.930	4.37 ± 0.25	4.41 ± 0.28	0.517	4.37 ± 0.26	4.54 ± 0.21	0.192	4.37 ± 0.26	4.47 ± 0.19	0.255	4.38 ± 0.27	4.39 ± 0.25	0.921	4.39 ± 0.24	4.37 ± 0.28	0.724	4.35 ± 0.24	4.39 ± 0.26	0.382
**iPTH (ng/mL)**	74.60 ± 30.76	67.74 ± 33.00	0.301	71.38 ± 34.28	62.17 ± 26.55	0.365	69.78 ± 32.90	56.08 ± 25.35	0.274	70.65 ± 33.35	57.80 ± 24.91	0.183	69.57 ± 31.10	68.33 ± 34.36	0.677	72.06 ± 32.63	63.94 ± 32.26	0.170	74.96 ± 31.42	66.84 ± 32.91	0.154
**25(OH)D_3_** **(total) (ng/mL)**	16.72 ± 8.30	18.85 ± 9.20	0.391	18.16 ± 8.71	19.35 ± 10.06	0.863	18.48 ± 9.02	18.24 ± 10.38	0.869	18.65 ± 9.08	17.26 ± 9.10	0.499	16.47 ± 8.06	20.57 ± 9.61	0.051	18.63 ± 9.68	18.20 ± 8.03	0.883	17.00 ± 9.89	18.99 ± 8.74	0.163
**GF (mL/min/1.73 m^2^)**	67.04 ± 16.55	71.63 ± 17.13	0.451	71.42 ± 17.45	69.11 ± 16.02	0.647	70.89 ± 17.35	69.56 ± 12.06	0.769	71.49 ± 17.03	66.28 ± 17.10	0.364	73.44 ± 18.32	68.05 ± 15.29	0.139	72.48 ± 17.77	68.10 ± 15.64	0.273	52.89 ± 15.21	77.17 ± 12.56	**<0.001**
	**Dynapenia**			**Mobility**			**Self-Care**			**Daily Activities**			**Pain/Discomfort**			**Anxiety/Depression**			**GFR**		
	**Low**	**Not Low**	***p***	**No Problem**	**Problem**	***p***	**No Problem**	**Problem**	***p***	**No Problem**	**Problem**	***p***	**No Problem**	**Problem**	***p***	**No Problem**	**Problem**	***p***	**1**	**2**	***p***
	**Mean ± SD**	**Mean ± SD**		**Mean ± SD**	**Mean ± SD**		**Mean ± SD**	**Mean ± SD**		**Mean ± SD**	**Mean ± SD**		**Mean ± SD**	**Mean ± SD**		**Mean ± SD**	**Mean ± SD**		**Mean ± SD**	**Mean ± SD**	
(**b**)																					
**Age**	80.56 ± 7.02	75.90 ± 6.82	**0.028**	75.92 ± 7.22	80.80 ± 5.36	**0.011**	76.79 ± 7.07	85.00 ± 1.41	0.078	76.79 ± 7.07	85.00 ± 1.41	0.078	77.11 ± 6.48	76.97 ± 7.89	0.895	76.39 ± 6.48	79.06 ± 8.68	0.293	80.74 ± 5.74	75.52 ± 7.11	**0.005**
**BMI**	28.22 ± 4.05	27.59 ± 3.94	0.589	27.65 ± 3.81	28.04 ± 4.48	0.840	27.62 ± 3.87	31.62 ± 6.05	0.262	27.62 ± 3.87	31.62 ± 6.05	0.262	27.12 ± 3.88	28.46 ± 3.96	0.184	28.31 ± 3.95	26.00 ± 3.49	0.070	28.91 ± 4.15	27.26 ± 3.79	0.171
**PASE**	264.38 ± 132.67	272.73 ± 182.18	0.709	294.24 ± 179.77	192.14 ± 104.97	**0.045**	273.16 ± 171.23	192.50 ± 166.17	0.487	273.16 ± 171.23	192.50 ± 166.17	0.487	320.61 ± 182.95	212.43 ± 135.31	**0.011**	296.81 ± 179.45	190.65 ± 108.43	**0.021**	267.96 ± 190.76	271.80 ± 163.49	0.0686
**Grip test right**	19.78 ± 3.73	54.21 ± 21.13	**<0.001**	46.56 ± 23.00	43.00 ± 26.56	0.483	45.60 ± 24.06	50.00 ± 0.00	0.554	45.60 ± 24.06	50.00 ± 0.00	0.554	46.83 ± 25.61	44.47 ± 21.62	0.911	43.76 ± 22.80	51.81 ± 26.09	0.225	49.63 ± 20.25	44.13 ± 25.01	0.194
**Grip test left**	18.97 ± 5.17	48.58 ± 20.81	**<0.001**	42.39 ± 21.90	37.63 ± 23.91	0.315	41.02 ± 22.51	50.00 ± 14.14	0.424	41.02 ± 22.51	50.00 ± 14.14	0.424	41.19 ± 22.17	41.42 ± 22.79	0.984	39.40 ± 21.53	47.09 ± 24.23	0.209	45.26 ± 19.50	39.65 ± 23.34	0.159
**EQ-5D**	0.81 ± 0.12	0.84 ± 0.18	0.235	0.89 ± 0.11	0.64 ± 0.16	**<0.001**	0.84 ± 0.14	0.43 ± 0.29	**0.004**	0.84 ± 0.14	0.43 ± 0.29	**0.004**	0.93 ± 0.11	0.71 ± 0.14	**<0.001**	0.88 ± 0.14	0.69 ± 0.16	**<0.001**	0.78 ± 0.21	0.85 ± 0.14	0.273
**EQ-VAS (1–100)**	70.31 ± 13.22	75.92 ± 13.45	0.137	76.50 ± 12.79	68.00 ± 14.24	**0.048**	75.48 ± 12.60	45.00 ± 7.07	**0.006**	75.48 ± 12.60	45.00 ± 7.07	**0.006**	76.00 ± 13.44	72.83 ± 13.63	0.374	75.82 ± 13.00	70.63 ± 14.71	0.193	73.16 ± 15.74	75.11 ± 12.63	0.707
**VAS (1–10)**	3.81 ± 2.76	2.19 ± 2.41	**0.035**	2.07 ± 2.22	4.33 ± 2.99	**0.009**	2.49 ± 2.55	5.75 ± 1.77	0.096	2.49 ± 2.55	5.75 ± 1.77	0.096	1.03 ± 1.38	4.42 ± 2.46	**<0.001**	2.60 ± 2.55	2.56 ± 2.76	0.883	2.66 ± 3.11	2.57 ± 2.36	0.849
**MNA**	13.63 ± 2.00	13.60 ± 1.92	0.944	13.33 ± 2.02	14.53 ± 1.19	**0.048**	13.60 ± 1.95	14.00 ± 1.41	0.894	13.60 ± 1.95	14.00 ± 1.41	0.894	13.61 ± 1.97	13.60 ± 1.90	0.973	13.36 ± 2.02	14.38 ± 1.41	0.076	14.00 ± 1.53	13.45 ± 2.06	0.403
**mtDNA-CN**	127.84 ± 39.49	131.51 ± 63.22	0.831	134.67 ± 61.25	117.06 ± 44.72	0.464	129.27 ± 58.09	172.80 ± 49.82	0.216	129.27 ± 58.09	172.80 ± 49.82	0.216	136.82 ± 63.35	123.36 ± 51.19	0.713	136.96 ± 56.99	111.14 ± 58.58	0.072	111.65 ± 56.83	138.44 ± 57.28	**0.031**
**Creatinine (ng/mL)**	1.00 ± 0.17	1.12 ± 0.29	0.072	1.08 ± 0.28	1.13 ± 0.23	0.366	1.08 ± 0.26	1.50 ± 0.18	**0.029**	1.08 ± 0.26	1.50 ± 0.18	**0.029**	1.06 ± 0.27	1.13 ± 0.27	0.269	1.06 ± 0.19	1.21 ± 0.42	0.589	1.37 ± 0.32	0.98 ± 0.14	**<0.001**
**Calcium (ng/mL)**	9.33 ± 0.32	9.41 ± 0.27	0.570	9.39 ± 0.29	9.39 ± 0.27	0.937	9.39 ± 0.28	9.35 ± 0.21	0.727	9.39 ± 0.28	9.35 ± 0.21	0.727	9.38 ± 0.27	9.40 ± 0.30	0.725	9.40 ± 0.27	9.38 ± 0.33	0.656	9.35 ± 0.31	9.41 ± 0.27	0.485
**Phosphorus (ng/mL)**	2.96 ± 0.52	3.13 ± 0.43	0.162	3.09 ± 0.47	3.10 ± 0.43	0.833	3.07 ± 0.45	3.75 ± 0.35	0.062	3.07 ± 0.45	3.75 ± 0.35	0.062	3.19 ± 0.46	2.98 ± 0.43	**0.028**	3.11 ± 0.45	3.03 ± 0.49	0.445	3.16 ± 0.41	3.07 ± 0.48	0.397
**Albumin (ng/mL)**	4.40 ± 0.28	4.47 ± 0.23	0.365	4.46 ± 0.24	4.43 ± 0.23	0.540	4.46 ± 0.24	4.25 ± 0.35	0.385	4.46 ± 0.24	4.25 ± 0.35	0.385	4.43 ± 0.26	4.47 ± 0.22	0.490	4.46 ± 0.23	4.44 ± 0.27	0.945	4.38 ± 0.20	4.48 ± 0.25	0.103
**iPTH (ng/mL)**	59.31 ± 22.09	62.90 ± 25.33	0.837	59.20 ± 23.82	71.41 ± 25.12	**0.029**	60.85 ± 23.87	99.62 ± 10.44	**0.030**	60.85 ± 23.87	99.62 ± 10.44	**0.030**	59.06 ± 21.37	65.46 ± 27.58	0.350	59.80 ± 24.91	68.83 ± 22.56	0.141	74.42 ± 29.34	56.84 ± 20.32	**0.030**
**25(OH)D_3_ (total) (ng/mL)**	15.01 ± 4.31	20.21 ± 10.60	0.070	19.76 ± 9.77	16.14 ± 9.13	0.055	18.78 ± 9.77	23.69 ± 5.65	0.231	18.78 ± 9.77	23.69 ± 5.65	0.231	19.97 ± 11.25	17.70 ± 7.45	0.571	19.88 ± 10.35	16.00 ± 6.71	0.117	21.79 ± 14.90	17.74 ± 6.29	0.880
**GFR (mL/min/1.73 m^2^)**	71.61 ± 11.46	66.72 ± 16.15	0.297	69.28 ± 15.39	63.40 ± 14.06	0.129	68.73 ± 14.69	42.45 ± 6.48	**0.024**	68.73 ± 14.69	42.45 ± 6.48	**0.024**	69.40 ± 13.87	66.20 ± 16.67	0.465	69.42 ± 12.53	63.33 ± 21.27	0.469	51.13 ± 12.82	74.86 ± 9.71	**<0.001**

SD: Standard Deviation; BMI: body mass index; PASE: Physical Activity Scale for the Elderly; EQ-5D: EuroQoL Five-Dimension Questionnaire; EQ-VAS: EQ-5D Visual Analogue Scale; VAS: Visual Analogue Scale; MNA: Mini Nutritional Assessment; mtDNA-CN: mitochondrial DNA copy number; iPTH: intact parathyroid hormone; 25(OH)D_3_: 25-hydroxyvitamin D_3_; GFR: Glomerular Filtration Rate. Dynapenia was considered low under 16 kg for women and under 27 kg for men. Statistically and marginally significant values are in bold.

**Table 4:** Association between dynapenia, quality of life dimensions, renal function and mitochondrial DNA copy number.

			Dynapenia		Mobility		Self-Care		Daily Activities		Pain/Discomfort		Anxiety/Depression		**Glomerular Filtration**	
			OR (95% CI)	*p*	OR (95% CI)	*p*	OR (95% CI)	*p*	OR (95% CI)	*p*	OR (95% CI)	*p*	OR (95% CI)	*p*	**OR (95% CI)**	***p***
**Total**	**Univariate**	**mtDNA-CN**	1.005 (1.00–1.01)	0.164	0.991 (0.98–1.00)	**0.017**	0.993 (0.99–1.01)	0.357	0.998 (0.99–1.01)	0.625	0.997 (0.99–1.00)	0.344	0.998 (0.99–1.00)	0.477	1.004 (1.00–1.01)	0.257
		**Age**	0.940 (0.89–1.00)	**0.032**	1.053 (1.00–1.11)	0.057	0.994 (0.89–1.11)	0.916	1.021 (0.94–1.11)	0.610	1.011 (0.97–1.06)	0.652	1.038 (0.99–1.09)	0.134	0.894 (0.85–0.95)	**<0.001**
		**PASE**	1.000 (0.99–1.00)	0.686	0.994 (0.99–1.00)	**<0.001**	0.997 (0.99–1.00)	0.249	0.997 (0.99–1.00)	0.210	0.996 (0.99–1.00)	**<0.001**	0.995 (0.99–1.00)	**<0.001**	1.001 (0.99–1.00)	0.375
	**Multivariate**	**mtDNA-CN**	1.005 (1.00–1.01)	0.206	0.991 (0.98–1.00)	**0.022**	0.994 (0.98–1.01)	0.392	0.998 (0.99–1.01)	0.691	0.998 (0.99–1.00)	0.458	0.999 (0.99–1.01)	0.655	1.003 (1.00–1.01)	0.398
		**Age**	0.942 (0.89–1.00)	**0.039**	1.055 (1.00–1.12)	0.069	0.992 (0.89–1.10)	0.877	1.018 (0.94–1.10)	0.665	1.004 (0.96–1.05)	0.856	1.036 (0.98–1.09)	0.180	0.896 (0.85–0.95)	**<0.001**
		**PASE**	0.999 (0.99–1.00)	0.553	0.993 (0.99–1.00)	**<0.001**	0.997 (0.99–1.00)	0.268	0.997 (0.99–1.00)	0.224	0.996 (0.99–1.00)	**<0.001**	0.995 (0.99–1.00)	**<0.001**	1.001 (0.99–1.00)	0.490
**Women**	**Univariate**	**mtDNA-CN**	1.009 (0.99–1.02)	0.124	0.987 (0.98–1.00)	**0.022**	0.976 (0.95–1.00)	0.068	0.992 (0.98–1.01)	0.226	0.998 (0.99–1.01)	0.596	1.000 (0.99–1.01)	0.961	1.000 (0.99–1.01)	0.967
		**Age**	0.973 (0.90–1.05)	0.496	1.021 (0.95–1.09)	0.547	0.922 (0.79–1.07)	0.292	1.003 (0.92–1.10)	0.944	1.023 (0.96–1.09)	0.472	1.037 (0.97–1.10)	0.262	0.896 (0.83–0.97)	**0.004**
		**PASE**	0.999 (0.99–1.00)	0.423	0.992 (0.99–1.00)	**0.001**	0.997 (0.99–1.00)	0.319	0.997 (0.99–1.00)	0.230	0.996 (0.99–1.00)	**0.008**	0.995 (0.99–1.00)	**0.003**	1.002 (1.00–1.01)	0.271
	**Multivariate**	**mtDNA-CN**	1.009 (1.00–1.02)	0.138	0.983 (0.97–1.00)	**0.009**	0.970 (0.94–1.00)	0.067	0.992 (0.98–1.00)	0.203	0.998 (0.99–1.00)	0.537	1.000 (0.99–1.00)	0.909	1.000 (0.99–1.01)	0.918
		**Age**	0.975 (0.90–1.06)	0.524	1.022 (0.95–1.10)	0.579	0.904 (0.77–1.07)	0.232	1.000 (0.91–1.09)	0.993	1.020 (0.96–1.09)	0.550	1.036 (0.97–1.01)	0.298	0.896 (0.83–0.97)	**0.005**
		**PASE**	0.999 (0.99–1.00)	0.445	0.991 (0.99–1.00)	**<0.001**	0.996 (0.99–1.00)	0.239	0.997 (0.99–1.00)	0.206	0.996 (0.99–1.00)	**0.008**	0.995 (0.99–1.00)	**0.003**	1.002 (1.00–1.01)	0.316
**Men**	**Univariate**	**mtDNA-CN**	1.001 (0.99–1.01)	0.825	0.994 (0.98–1.01)	0.304	1.011 (0.99–1.03)	0.310	1.011 (0.99–1.03)	0.310	0.996 (0.99–1.01)	0.351	0.991 (0.98–1.00)	0.127	1.009 (1.00–1.02)	0.096
		**Age**	0.906 (0.83–0.99)	**0.028**	1.110 (1.01–1.22)	**0.025**	1.202 (0.94–1.53)	0.140	1.202 (0.94–1.53)	0.140	0.997 (0.93–1.07)	0.933	1.056 (0.97–1.15)	0.194	0.893 (0.82–0.97)	**0.010**
		**PASE**	1.000 (0.99–1.00)	0.864	0.995 (0.99–1.00)	**0.050**	0.996 (0.99–1.01)	0.516	0.996 (0.99–1.01)	0.516	0996 (0.99–1.00)	**0.015**	0.995 (0.99–1.00)	**0.038**	1.000 (0.99–1.00)	0.934
	**Multivariate**	**mtDNA-CN**	1.000 (0.99–1.01)	0.995	0.995 (0.98–1.00)	0.463	1.036 (0.98–1.10)	0.236	1.036 (0.98–1.10)	0.236	0.998 (0.99–1.01)	0.612	0.992 (0.98–1.01)	0.218	1.010 (1.00–1.02)	0.114
		**Age**	0.906 (0.83–0.99)	**0.029**	1.118 (1.01–1.23)	**0.025**	1.520 (0.77–2.98)	0.224	1.520 (0.77–2.98)	0.224	0.988 (0.92–1.06)	0.744	1.059 (0.97–1.16)	0.202	0.892 (0.82–0.98)	**0.013**
		**PASE**	1.000 (0.99–1.00)	0.977	0.995 (0.99–1.00)	0.055	0.994 (0.98–1.01)	0.421	0.994 (0.98–1.01)	0.421	0.996 (0.99–1.00)	**0.018**	0.995 (0.99–1.00)	0.052	1.000 (0.99–1.00)	0.807

OR: Odds Ratio; CI: Confidence Interval; mtDNA-CN: mitochondrial DNA copy number; PASE: Physical Activity Scale for the Elderly. Dynapenia was considered low under 16 kg for women and under 27 kg for men. Statistically and marginally significant values are in bold.

## Data Availability

The data presented in this study are available on request from the corresponding author. The data are not publicly available due to privacy and ethical restrictions.

## Abbreviations

The following abbreviations are used in this manuscript:

25(OH)D_3_	25-hydroxyvitmin D_3_
Alb	Albumin
BMD	Bone mineral density
CGR	Clinical group risk
CI	Confidence interval
CKD-EPI	Chronic Kidney Disease Epidemiology Collaboration
CVD	Cardiovascular disease
ECLIA	Electrochemiluminescent immunoassay
EQ-5D	EuroQoL 5-dimension questionnaire
EQ-VAS	EQ-5D visual analogue scale
GFR	Glomerular filtration rate
HGS	Hand grip strength
iPTH	Intact parathyroid hormone
MNA	Mini Nutritional Assessment
mtDNA	Mitochondrial DNA
mtDNA-CN	Mitochondrial DNA copy number
OR	Odds ratio
OXPHOS	Oxidative phosphorylation
PASE	Physical Activity Scale for the Elderly
PBMCs	Peripheral blood mononuclear cells
QoL	Quality of life
qPCR	Quantitative real-time PCR
ROS	Reactive oxygen species
SACyL	Castilla y León National Health System
SD	Standard deviation
VAS	Visual analogue scale
VDR	Vitamin D receptor
χ^2^ test	Chi-square test

## References

[1] Voet D.V.J., Pratt C.W., Jain S.L., Chand S. (2005). Fundamentals of Biochemistry.

[2] Osellame L.D., Blacker T.S., Duchen M.R. (2012). Cellular and Molecular Mechanisms of Mitochondrial Function. Best Pract. Res. Clin. Endocrinol. Metab..

[3] Arciuch V.G.A., Elguero M.E., Poderoso J.J., Carreras M.C. (2012). Mitochondrial Regulation of Cell Cycle and Proliferation. Antioxid. Redox Signal.

[4] Miller F.J., Rosenfeldt F.L., Zhang C., Linnane A.W., Nagley P. (2003). Precise Determination of Mitochondrial DNA Copy Number in Human Skeletal and Cardiac Muscle by a PCR-Based Assay: Lack of Change of Copy Number with Age. Nucleic Acids Res..

[5] Seo A.Y., Joseph A.M., Dutta D., Hwang J.C.Y., Aris J.P., Leeuwenburgh C. (2010). New Insights into the Role of Mitochondria in Aging: Mitochondrial Dynamics and More. J. Cell Sci..

[6] John J.C.S. (2016). Mitochondrial DNA Copy Number and Replication in Reprogramming and Differentiation. Semin. Cell Dev. Biol..

[7] Jeng J.Y., Yeh T.S., Lee J.W., Lin S.H., Fong T.H., Hsieh R.H. (2008). Maintenance of Mitochondrial DNA Copy Number and Expression Are Essential for Preservation of Mitochondrial Function and Cell Growth. J. Cell Biochem..

[8] Coen P.M., Musci R.V., Hinkley J.M., Miller B.F. (2019). Mitochondria as a Target for Mitigating Sarcopenia. Front. Physiol..

[9] Czajka A., Ajaz S., Gnudi L., Parsade C.K., Jones P., Reid F., Malik A.N. (2015). Altered Mitochondrial Function, Mitochondrial DNA and Reduced Metabolic Flexibility in Patients with Diabetic Nephropathy. eBioMedicine.

[10] Kramer P.A., Ravi S., Chacko B., Johnson M.S., Darley-Usmar V.M. (2014). A Review of the Mitochondrial and Glycolytic Metabolism in Human Platelets and Leukocytes: Implications for Their Use as Bioenergetic Biomarkers. Redox Biol..

[11] Rose S., Carvalho E., Diaz E.C., Cotter M., Bennuri S.C., Azhar G., Frye R.E., Adams S.H., Børsheim E. (2019). A Comparative Study of Mitochondrial Respiration in Circulating Blood Cells and Skeletal Muscle Fibers in Women. Am. J. Physiol. Endocrinol. Metab..

[12] Jafari Z., Kolb B.E., Mohajerani M.H. (2021). Age-Related Hearing Loss and Cognitive Decline: MRI and Cellular Evidence. Ann. N. Y. Acad. Sci..

[13] Wang S., Yang Y., Pang Z., Li Y., Li K., Sun Y., Yang J. (2025). Mitochondrial DNA Copy Number as a Genetic Determinant of Renal Function: Insights from Bidirectional Mendelian Randomization. Ren. Fail..

[14] Wu I.C., Lin C.C., Liu C.S., Hsu C.C., Chen C.Y., Hsiung C.A. (2017). Interrelations between Mitochondrial DNA Copy Number and Inflammation in Older Adults. J. Gerontol. A Biol. Sci. Med. Sci..

[15] Ashar F.N., Moes A., Moore A.Z., Grove M.L., Chaves P.H.M., Coresh J., Newman A.B., Matteini A.M., Bandeen-Roche K., Boerwinkle E. (2015). Association of Mitochondrial DNA Levels with Frailty and All-Cause Mortality. J. Mol. Med..

[16] Mengel-From J., Thinggaard M., Dalgård C., Kyvik K.O., Christensen K., Christiansen L. (2014). Mitochondrial DNA Copy Number in Peripheral Blood Cells Declines with Age and Is Associated with General Health among Elderly. Hum. Genet..

[17] Knez J., Marrachelli V.G., Cauwenberghs N., Winckelmans E., Zhang Z., Thijs L., Brguljan-Hitij J., Plusquin M., Delles C., Monleon D. (2017). Peripheral Blood Mitochondrial DNA Content in Relation to Circulating Metabolites and Inflammatory Markers: A Population Study. PLoS ONE.

[18] Haussler M.R., Whitfield G.K., Kaneko I., Haussler C.A., Hsieh D., Hsieh J.C., Jurutka P.W. (2013). Molecular Mechanisms of Vitamin D Action. Calcif. Tissue Int..

[19] Dursun E., Gezen-Ak D., Yilmazer S. (2011). A Novel Perspective for Alzheimer’s Disease: Vitamin D Receptor Suppression by Amyloid-β and Preventing the Amyloid-β Induced Alterations by Vitamin D in Cortical Neurons. J. Alzheimer’s Dis..

[20] Giustina A., Bouillon R., Dawson-Hughes B., Ebeling P.R., Lazaretti-Castro M., Lips P., Marcocci C., Bilezikian J.P. (2023). Vitamin D in the Older Population: A Consensus Statement. Endocrine.

[21] Verde Z., Giaquinta A., Moreno Sainz C., Díaz Ondina M., Fernández Araque A. (2019). Bone Mineral Metabolism Status, Quality of Life, and Muscle Strength in Older People. Nutrients.

[22] Gezen-Ak D., Alaylıoğlu M., Yurttaş Z., Çamoğlu T., Şengül B., İşler C., Yaşar Kına Ü., Keskin E., Atasoy İ.L., Kafardar A.M. (2023). Vitamin D Receptor Regulates Transcription of Mitochondrial DNA and Directly Interacts with Mitochondrial DNA and TFAM. J. Nutr. Biochem..

[23] Kim J.H., Im J.A., Lee D.C. (2012). The Relationship between Leukocyte Mitochondrial DNA Contents and Metabolic Syndrome in Postmenopausal Women. Menopause.

[24] Hughes J.S., Averill R.F., Eisenhandler J., Goldfield N.I., Muldoon J., Neff J.M., Gay J.C. (2004). Clinical Risk Groups (CRGs) a Classification System for Risk-Adjusted Capitation-Based Payment and Health Care Management. Med. Care.

[25] Devlin N., Pickard S., Busschbach J. (2022). The Development of the EQ-5D-5L and Its Value Sets for EQ-5D-5L: A Compendium, Comparative Review & User Guide. Value Sets for EQ-5D-5L: A Compendium, Comparative Review & User Guide.

[26] EQ-5D-5L-EuroQol. https://euroqol.org/information-and-support/euroqol-instruments/eq-5d-5l/.

[27] Washburn R.A., Smith K.W., Jette A.M., Janney C.A. (1993). The Physical Activity Scale for the Elderly (PASE): Development and Evaluation. J. Clin. Epidemiol..

[28] Washburn R.A., McAuley E., Katula J., Mihalko S.L., Boileau R.A. (1999). The Physical Activity Scale for the Elderly (PASE): Evidence for Validity. J. Clin. Epidemiol..

[29] Kammar-García A., Garza-Santiago E., Mancilla-Galindo J., Segura-Badilla O.L., Lazcano-Hernández M., Vera-López O., Navarro-Cruz A.R. (2025). Usefulness of the Mini-Nutritional Assessment in Screening for Sarcopenia in a Sample of Institutionalized Older Persons—A Cross-Sectional Study. Nutr. Hosp..

[30] Vellas B., Guigoz Y., Garry P.J., Nourhashemi F., Bennahum D., Lauque S., Albarede J.L. (1999). The Mini Nutritional Assessment (MNA) and Its Use in Grading the Nutritional State of Elderly Patients. Nutrition.

[31] Cruz-Jentoft A.J., Bahat G., Bauer J., Boirie Y., Bruyère O., Cederholm T., Cooper C., Landi F., Rolland Y., Sayer A.A. (2019). Sarcopenia: Revised European Consensus on Definition and Diagnosis. Age Ageing.

[32] Semergen Cantabria. http://www.semergencantabria.org/calculadoras.htm?17.

[33] Venegas V., Halberg M.C. (2012). Measurement of Mitochondrial DNA Copy Number. Methods Mol. Biol..

[34] Picca A., Calvani R., Bossola M., Allocca E., Menghi A., Pesce V., Lezza A.M.S., Bernabei R., Landi F., Marzetti E. (2018). Update on Mitochondria and Muscle Aging: All Wrong Roads Lead to Sarcopenia. Biol. Chem..

[35] Wachsmuth M., Hübner A., Li M., Madea B., Stoneking M. (2016). Age-Related and Heteroplasmy-Related Variation in Human MtDNA Copy Number. PLoS Genet..

[36] Hebert S.L., Marquet-De Rougé P., Lanza I.R., McCrady-Spitzer S.K., Levine J.A., Middha S., Carter R.E., Klaus K.A., Therneau T.M., Highsmith E.W. (2015). Mitochondrial Aging and Physical Decline: Insights from Three Generations of Women. J. Gerontol. A Biol. Sci. Med. Sci..

[37] Kim J.H., Lee D.C. (2012). Mitochondrial DNA Copy Number in Peripheral Blood Is Associated with Femoral Neck Bone Mineral Density in Postmenopausal Women. J. Rheumatol..

[38] DeLuca H.F. (2004). Overview of General Physiologic Features and Functions of Vitamin D. Am. J. Clin. Nutr..

[39] Ryan Z.C., Craig T.A., Folmes C.D., Wang X., Lanza I.R., Schaible N.S., Salisbury J.L., Nair K.S., Terzic A., Sieck G.C. (2015). 1α,25-Dihydroxyvitamin D3 Regulates Mitochondrial Oxygen Consumption and Dynamics in Human Skeletal Muscle Cells. J. Biol. Chem..

[40] Takahashi Y., Hijikata K., Seike K., Nakano S., Banjo M., Sato Y., Takahashi K., Hatta H. (2018). Effects of Royal Jelly Administration on Endurance Training-Induced Mitochondrial Adaptations in Skeletal Muscle. Nutrients.

[41] Herbst A., Prior S.J., Lee C.C., Aiken J.M., McKenzie D., Hoang A., Liu N., Chen X., Xun P., Allison D.B. (2021). Skeletal Muscle Mitochondrial DNA Copy Number and Mitochondrial DNA Deletion Mutation Frequency as Predictors of Physical Performance in Older Men and Women. Geroscience.

[42] Fan Z., Yang J.Y., Guo Y., Liu Y.X., Zhong X.Y. (2022). Altered Levels of Circulating Mitochondrial DNA in Elderly People with Sarcopenia: Association with Mitochondrial Impairment. Exp. Gerontol..

[43] Fábrega-Cuadros R., Hita-Contreras F., Martínez-Amat A., Jiménez-García J.D., Achalandabaso-Ochoa A., Lavilla-Lerma L., García-Garro P.A., Álvarez-Salvago F., Aibar-Almazán A. (2021). Associations between the Severity of Sarcopenia and Health-Related Quality of Life in Community-Dwelling Middle-Aged and Older Adults. Int. J. Environ. Res. Public Health.

[44] Beaudart C., Demonceau C., Reginster J.Y., Locquet M., Cesari M., Cruz Jentoft A.J., Bruyère O. (2023). Sarcopenia and Health-Related Quality of Life: A Systematic Review and Meta-Analysis. J. Cachexia Sarcopenia Muscle.

[45] Shigehara K., Kato Y., Izumi K., Mizokami A. (2022). Relationship between Testosterone and Sarcopenia in Older-Adult Men: A Narrative Review. J. Clin. Med..

[46] Geraci A., Calvani R., Ferri E., Marzetti E., Arosio B., Cesari M. (2021). Sarcopenia and Menopause: The Role of Estradiol. Front. Endocrinol..

[47] Kim Y.J., Tamadon A., Park H.T., Kim H., Ku S.-Y. (2016). The Role of Sex Steroid Hormones in the Pathophysiology and Treatment of Sarcopenia. Osteoporos. Sarcopenia.

[48] Kramer P.A., Coen P.M., Cawthon P.M., Distefano G., Cummings S.R., Goodpaster B.H., Hepple R.T., Kritchevsky S.B., Shankland E.G., Marcinek D.J. (2024). Skeletal Muscle Energetics Explain the Sex Disparity in Mobility Impairment in the Study of Muscle, Mobility and Aging. J. Gerontol. A Biol. Sci. Med. Sci..

[49] Triolo M., Oliveira A.N., Kumari R., Hood D.A. (2022). The Influence of Age, Sex, and Exercise on Autophagy, Mitophagy, and Lysosome Biogenesis in Skeletal Muscle. Skelet. Muscle.

[50] Moreira-Pais A., Vitorino R., Sousa-Mendes C., Neuparth M.J., Nuccio A., Luparello C., Attanzio A., Novák P., Loginov D., Nogueira-Ferreira R. (2024). Mitochondrial Remodeling Underlying Age-Induced Skeletal Muscle Wasting: Let’s Talk about Sex. Free Radic. Biol. Med..

[51] Che R., Yuan Y., Huang S., Zhang A. (2014). Mitochondrial Dysfunction in the Pathophysiology of Renal Diseases. Am. J. Physiol. Ren. Physiol..

[52] Forbes J.M. (2016). Mitochondria-Power Players in Kidney Function?. Trends Endocrinol. Metab..

[53] Braga P.C., Alves M.G., Rodrigues A.S., Oliveira P.F. (2022). Mitochondrial Pathophysiology on Chronic Kidney Disease. Int. J. Mol. Sci..

[54] Yan X., Yang P., Li Y., Liu T., Zha Y., Wang T., Zhang J., Feng Z., Li M. (2024). New insights from bidirectional Mendelian randomization: Causal relationships between telomere length and mitochondrial DNA copy number in aging biomarkers. Aging.

[55] Ashar F.N., Zhang Y., Longchamps R.J., Lane J., Moes A., Grove M.L., Mychaleckyj J.C., Taylor K.D., Coresh J., Rotter J.I. (2017). Association of Mitochondrial DNA Copy Number with Cardiovascular Disease. JAMA Cardiol..

